# Loss with ageing but preservation of frontal cortical capillary pericytes in post-stroke dementia, vascular dementia and Alzheimer’s disease

**DOI:** 10.1186/s40478-021-01230-6

**Published:** 2021-08-02

**Authors:** Ren Ding, Yoshiki Hase, Matthew Burke, Vincent Foster, William Stevenson, Tuomo Polvikoski, Raj N. Kalaria

**Affiliations:** Neurovascular Research Group, Translational and Clinical Research Institute, Newcastle University, Campus for Ageing and Vitality, Newcastle Upon Tyne, NE4 5PL UK

**Keywords:** Alzheimer's disease, Cerebral cortex, Collagen IV, Dementia, Pericyte, Platelet derived growth factor receptor, Post-stroke dementia, Vascular dementia

## Abstract

Cerebral pericytes are an integral component of the neurovascular unit, which governs the blood–brain barrier. There is paucity of knowledge on cortical pericytes across different dementias. We quantified cortical pericytes in capillaries in 124 post-mortem brains from subjects with post-stroke dementia (PSD), vascular dementia (VaD), Alzheimer’s disease (AD) and AD-VaD (Mixed) and, post-stroke non-demented (PSND) stroke survivors as well as normal ageing controls. Collagen 4 (COL4)-positive nucleated pericyte soma were identified as protrusions on capillaries of the frontal cortex. The COL4-positive somata or nodule-like cell bodies were also verified by platelet derived growth factor receptor-β (PDGFR-β) immunohistochemistry. The mean (± SEM) pericyte somata in frontal cortical capillaries in normal young controls (46–65 years of age) was estimated as 5.2 ± 0.2 per mm capillary length. This number was reduced by 45% in older controls (> 78 years) to 2.9 ± 0.1 per mm capillary length (*P* < 0.001). We further found that the numbers of pericyte cell bodies per COL4 mm^2^ area or per mm capillary length were not decreased but rather preserved or increased in PSD, AD and Mixed dementia groups compared to similar age older controls (*P* < 0.01). Consistent with this, we noted that capillary length densities identified by the endothelial marker glucose transporter 1 or COL4 were not different across the dementias compared to older controls. There was a negative correlation with age (*P* < 0.001) suggesting fewer pericyte somata in older age, although the % COL4 immunoreactive capillary area was increased in older controls compared to young controls. Using a proven reliable method to quantify COL4-positive nucleated pericytes, our observations demonstrate ageing related loss but mostly preserved pericytes in the frontal cortex of vascular and AD dementias. We suggest there is differential regulation of capillary pericytes in the frontal lobe between the cortex and white matter in ageing-related dementias.

**Supplementary Information:**

The online version contains supplementary material available at 10.1186/s40478-021-01230-6.

## Background

Brain pericytes have become of increased interest in health and disease. Pericytes are pluripotent cells with characteristic phenotype, distribution and function [6, 7, 15, 21]. Pericytes form one of the key cellular components of the neurovascular unit, which controls the blood–brain barrier (BBB). Experimental evidence suggests pericytes regulate BBB integrity by differentially expressing proteins such as α-smooth muscle actin [47]. The platelet-derived growth factor (PDGF) is one of the major signalling pathways identified in pericytes [29] that has been used to monitor pericyte pathophysiology in several neurological diseases including dementia [21, 31]. Soluble PDGF-β released from endothelial cells recruits pericytes via the PDGF receptor-β (PDGFR-β) and disruption of PDGFR-β signalling results in fewer recruited pericytes to the vessel, causing vessel leakage, tortuosity, microaneurysms, and microbleeds [10]. In addition to PDGFR-β, a proportion of pericytes also express the bone morphogenetic protein-4 (BMP4) [48] that can be altered in cerebral small vessel disease. BMP4 expression is upregulated during cerebral hypoperfusion to promote angiogenesis but induces astrogliogenesis at the expense of other cells such as oligodendrocyte precursor cell proliferation [48]. In the hypoxic environment, pericytes may adopt microglial phenotypes and upregulate reactive cell responses [41]. Experimental studies also suggest while pericyte somata can be immobile, the tips of their processes undergo extensions and retractions over prolonged periods of time [5]. This indicates pericytes are highly ‘plastic’ and pericyte coverage may readily change during different homeostatic conditions. Furthermore, acute ablation of cortical pericytes may lead to rapid neurovascular uncoupling [32]. These observations also support the role of pericytes in cerebral blood flow (CBF) regulation and collectively have implications for neurological conditions associated with rapid pericyte loss such as cerebral hypoperfusion and stroke, as well as conditions where the time course of regional pericyte loss is less clear, such as Alzheimer's disease (AD) and other neurogenerative disorders [51].

Previous studies have indicated pericytes or their coverage specifically alter during neurodegenerative and vascular disease processes [16, 34, 36, 39, 40, 49]. In the parietal cortex of AD subjects, loss of PDGFR-β immunoreactivity was associated with fibrinogen leakage and reduced oxygenation, and related to fibrillar amyloid β accumulation [34]. Direct capillary constriction by amyloid β at perivascular sites was proposed to reduce energy lack and neurodegeneration in AD [40]. However, a very recent study [18], further reported that the pericyte population was maintained in tandem with increased capillary density in the frontal cortex of AD subjects. The investigators suggested that pericyte loss at least in the frontal cortex was not a general hallmark of AD pathology.

We previously reported that collagen 4 (COL4)-positive pericytes in the frontal deep white matter were markedly decreased across different dementias including AD, Mixed AD-vascular dementia (VaD) as well as post-stroke dementia (PSD) and VaD subjects [16]. Pericyte numbers were correlated with PDGFR-β reactivity and associated with damage to the BBB as indicated by disintegration of the gliovascular unit incorporating clasmatodendrosis of perivascular astrocytes [11, 23]. However, it is not clear how cerebral pericytes in the cerebral cortex above alter in different common dementias. Here, we used a previously validated method to identify nucleated pericytes and determined their status across different neurocognitive disorders including PSD, VaD, AD and mixed dementia with Alzheimer and vascular pathologies. Given their highly plastic nature, we tested the hypothesis that pericytes are differentially regulated in neurodegenerative and vascular diseases. We mainly focussed on abluminal located pericyte somata on capillaries of the frontal cortex as key cellular elements of the neurovascular unit [22].

## Methods

### Human subjects

In total, we assessed brain tissues from 124 subjects enrolled in our longitudinal prospective studies on brain ageing and dementia. Dementia was diagnosed in life and confirmed at post-mortem examination as either AD, mixed AD-VaD, (Mixed), VaD or PSD. In addition, we assessed post-stroke no dementia (PSND) subjects enrolled in the Newcastle Cognitive Function After Stroke (CogFAST) study [2]. The VaD and PSD subjects were also from the CogFAST study [2]. We compared dementia subjects with young (46–65 years of age) and older controls (78–94 years) without any dementia causing neurological or psychiatric disorder and no neuropathological diagnosis. Controls subjects were participants either in previous prospective studies or from unrelated brain donations to the Newcastle Brain Tissue Resource (NBTR). The mean age of the older controls was not different from any of the dementia subjects but significantly greater in comparison to young controls (Table 1). Local research ethics committees of the Newcastle upon Tyne NHS Foundation Hospitals Trust granted ethical approvals. Permission to use brains for post-mortem research was also granted by consent from the individuals themselves when they had been still alive or next-of-kin or family member. All brain tissues were retained in the NBTR.

**Table 1:** Demographic details of all the cases and controls

Variable	Young Controls	Older Controls	PSND	PSD	VaD	AD	Mixed
N	12	20	21	20	17	16	18
Mean Age, years (range)	57.5* (46–65)	79.3 (78–94)	85.1 (75–96)	87.1 (75–96)	84.2 (71–98)	84.2 (76–96)	85.1 (72–93)
Gender (M:F ratio)	55:45	35:65	57:43	30:70	41:59	56:44	44:56
MMSE, mean ± SEM	na	29 ± 1	27 ± 0.4	16 ± 1	13 ± 4	7 ± 2	11 ± 2
CAMCOG, mean ± SEM	na	na	90 ± 1	66 ± 3	na	39 ± 7	na
Braak Stage, mean (range)	0.25 (0–1)	1.9 (0–4)	2.6 (1–4)	2.6 (1–4)	2.0 (0–4)	5.6 (5–6) *	5.2 (5–6) *
CERAD, mean (range)	0.0 (0–0)	0.5 (0–2)	1.7 (1–2)	1.3 (1–3)	1.0 (0–2)	2.9 (2–3) *	2.9 (2–3) *
ABC Scores, mean	na	A0.5, B1.2, C0.5	A0.5, B1.2, C0.7	A0.5, B1.2, C0.8	A0.6, B1.2, C0.8	A3, B3, C3	A2.5, B2.6, C2.6
CAA frequency (moderate-severe), %	0%	6%	15%	18%	17%	39%	9%
Vascular pathology score, mean (range)†	na	6.7 (0–10) *	13.5 (13–14)	13.3 (9–17)	13.2 (10–16)	10.8 (3–16)	11.0 (6–14)
Cortical Infarct pathology (%)††	0%	0%	90%	90%	67%	41%	73%
WML score, mean (range)‡	na	0.5 (0–2) **	2.5 (2–3)	2.4 (2–3)	2.9 (2–3)	1.8 (0–3)	2.9 (2–3)
WM/ Vascular lesions, moderate—severe (%)‖	na	17.6%**	100%	100%	100%	72%	95%
Length density (L_v)_ of Cortical GLUT1 Capillaries (mm/mm^3^)⁋	na	0.72 ± 0.10	0.62 ± 0.17	0.78 ± 0.07	0.72 ± 0.09	0.72 ± 0.13	0.70 ± 0.10

Numbers represent mean values (± SEM) and where given with the range of values in parentheses. The causes of death included bronchopneumonia (95%), sudden cardiac arrest, carcinoma, renal failure, and gastrointestinal bleed with no distribution pattern in any group. The post-mortem interval between death and tissue retrieval ranged 24–47 h for all the cases. There were no differences in the length of post-mortem delay between groups. Mean age of young controls was different compared to older controls (*P < 0.05). Braak staging scores and Alzheimer’s Disease Neuropathologic changes [37] were different in mixed and AD cases compared to all other groups (*P < 0.05)

^†^Mean vascular pathology scores (range) derived as described previously [14] (*P < 0.05)

^††^Cortical infarct pathology includes small infarcts and microinfarcts in frontal and temporal lobes, designated as % was number of cases in which score was more than 4 (moderate to severe) [14]

^‡^WML Score, white matter pathology score assessed using the scale from [14]. Mean WML Score was high in all post-stroke and dementia subjects compared to controls (**P < 0.01)

‖WM/Vascular lesions, **P < 0.01 compared to all post-stroke and dementia subjects

⁋Determined at length density (L_v_) with GLUT1 as marker of capillaries [9, 22]. Abbreviations: ABC, AD Neuropathology scoring system; AD, Alzheimer’s disease; CAA, cerebral amyloid angiopathy; CAMCOG, Cambridge cognition examination; F, female; GLUT1, glucose transporter 1; M, male; MMSE, Mini Mental state examination; N, number of subjects; na, not available; NPD, no pathological diagnosis; PSND, post-stroke non-demented; PSD, post-stroke dementia; VaD, vascular dementia; WM, white matter; WML, white matter lesions

### Neuropathological examination and scoring

Neuropathological examination was carried out as described previously [24]. Tinctorial stains were used for assessment of structural integrity and infarcts, patterns of white matter attenuation and myelin loss and neuritic pathology. Amyloid-β immunohistochemistry for ABC rating of neuritic plaques, Gallays stain for neuritic pathology, and tau immunohistochemistry for Braak staging of neurofibrillary tangles. The clinical diagnosis of AD was confirmed on evidence of significant Alzheimer’s-type pathology incorporating Braak stages V–VI, moderate-severe CERAD [33] and high ABC scores per National Institute of Aging-Alzheimer’s Association guidelines [37] and an absence of significant vascular pathology. VaD diagnosed clinically, was confirmed by the presence of single, multiple or cystic infarcts, lacunes, border-zone infarcts, microinfarcts and arteriosclerosis and Braak stage ≤ IV [27, 28]. Mixed AD and VaD cases were classified in presence of high degrees of AD pathology (Braak V-VI) and significant vascular pathology (Table 1).

Vascular pathology scores were derived from the presence of vascular lesions/pathologies as described previously [14]. WM lesion (WML) scores were determined on scale of 0 to 3 signifying none, mild, moderate, and severe. Previously, we had shown there was 95% agreement in scoring between two assessors [14]. WM/vascular lesion severity was graded from low to severe in quartiles essentially as described previously [26]. Vascular indices were compatible with the recently established vascular cognitive impairment neuropathology consortium criteria [45]. Neuropathological examination was verified by TP and RNK. The entire morphological analyses were undertaken under operator-blinded conditions and samples were coded with sequential numbers. In addition, at least two of both positive and negative controls were included to monitor staining quality.

### Immunohistochemistry

We analysed formalin-fixed paraffin-embedded whole coronal sections at levels 6–8 [27, 42] containing the frontal cortex (Brodmann area 9). We ensured to select the cortical regions without any obvious infarction. Unless otherwise stated, 2–5 adjacent or alternate whole or half-coronal sections were used for the morphological analyses. Immunohistochemistry was performed to examine different microvascular structures essentially as described before [13, 22]. The following antibodies were used to assess various cellular features: collagen IV (COL4 at dilation 1:1000, C1926, Merck (Sigma-Aldrich), Branchburg, NJ, USA), a marker of basement membrane in the vessels, platelet-derived growth factor receptor-β (PDGFR-β at 1:200 dilution, clone 42G12, #AF385, R&D systems, Minneapolis, MN, USA), a marker for pericytes, bone morphogenetic protein 4 (BMP4 dilution at 1:100, MBA1049, Millipore, MA, USA), α-smooth muscle actin (αSMA at dilution 1:1000, Clone 1A4, Dako, Cambridge, UK), a marker for mural cells, and glucose transporter-1 (GLUT-1 at 1:200, PA1-21,041, Fisher Scientific, Waltham, MA, USA), a marker of endothelial cells. Vectastain ABC mouse kits (PK-6102, Vector Laboratories, Burlingame, CA, USA) and Diaminobenzidine were used for single or double immunohistochemistry. Haematoxylin counterstain was used for ease in localising regions of interest.

### Immunofluorescence methods

Tissue sections were first treated with 0.1 mg/ml protease and then incubated overnight at 4 °C with primary antibodies to anti-COL4 (C1926 Sigma) monoclonal antibody, anti-PDGFR-β, (1:200 dilution, AF385, R&D Systems), αSMA (1:500 dilution, Clone 1A4, Dako), glucose transporter-1 (GLUT-1, 1:200, Thermo Scientific). Sections were washed with PBS and further incubated with donkey anti-goat conjugated Alexa Fluor 594 (1:1000, A11058, Thermo Fisher Scientific, Waltham, MA, USA) and rabbit anti-mouse Alexa Fluor 488 (1:1000, A11059, Thermo Fisher Scientific). Sections were then washed in PBS before mounting in Vectashield with DAPI (H-1200, Vector Laboratories). Images were captured using a Leica TCS SP2 (upright) and Zeiss Spinning Disk (Invert) confocal microscopes as described previously [12].

### Pericyte soma quantification

In accord with our work on the white matter [16], we used COL4 immunohistochemistry as a readily applied method to determine densities of capillary pericytes in regions remote from obvious infarcts or capillary cerebral amyloid angiopathy (CAA). Two to 5 sections were immunostained with COL4 antibodies only or in combination with GLUT1 or αSMA and then usually counterstained lightly with haematoxylin. In COL4 immunostained sections, nucleated pericyte cell bodies characteristically recognised as protrusions or “bumps” were counted manually along capillary profiles from more than 2000 captured images. The total number of pericyte cell bodies were then determined for each case from 8–25 frames per case and then a mean number was calculated per case. We encountered 11–20 nucleated pericyte cell bodies in each image and this ensured a consistent counting method. In total, we counted over 3,000 pericytes involving > 1,500 images within each group comprising of at least 10 cases. The pericyte somata were counted only if it had the characteristic shape and there was a haematoxylin-stained nucleus within identified at × 40 magnification. Different from previous studies [36], our counts were limited to cell bodies rather than pericyte coverage of the processes or extensions. Our aim was to assess potential alterations in pericyte nuclei per se for as accurate as possible assessment of pericyte cells.

### Stereological analysis of length density

To measure length density (L_v_) of capillaries in 3 × 30 μm thick sections of the frontal cortex, we used the Stereologer2000 software (Stereologer, WV, USA) with the spherical probe ‘space ball’ option as described previously [9]. The operating system was connected to a Zeiss Axiolab microscope with a motorised stage (Prior Scientific, UK). The spherical probe with a diameter of 18 μm was selected to allow for section shrinkage and an appropriate guard volume. An outline was drawn denoting the area of interest, which corresponded to the relevant cortical region at low magnification (× 5). A digitally generated, equally spaced grid was overlaid and used to ensure random sampling within x and y axis of the reference area. L_v_ was then calculated by counting the number of intersections between the probe and the parameter –n this instance microvasculature (ΣQ), and the area of sampling probe (ΣA) (L_v_ = 2(ΣQ/ ΣA)) at × 100 magnification [38]. The number of intersections was used to estimate the L_v_ for each case.

### Cortical atrophy analysis

Cortical atrophy in PSD and PSND versus older control subjects was estimated using the method proposed by White and colleagues [20]. We used three markers to estimate atrophy of the cortical ribbon of Brodmann area 9: the ratio of brain weight to intracranial volume, the ratio of cortical thickness to head diameter, and neuronal loss. Brain weight were available from the records. Intracranial volume was measured from magnetic resonance scans [11]. Cortical thickness was taken from the sulcus of area 9 at 2.5 × objective. The raw data were converted into a Z score allowing for each individual marker to be compared to one another: Z = (x-u)/σ, where: X = raw score, U = mean, and σ = standard deviation. Each marker was assigned a percentage weight indicating the extent of influence of the Z score. Thus, brain weight vs. intracranial volume (50%), cortical thickness vs. head diameter (40%) and neuron density (10%). The final score for each subject group was the total Z score based on the above percentages.

### Image acquisition and analysis

Images of capillary beds or regions of interest (ROI) within the cortical ribbon were captured on a Zeiss Axioplan 2.0 microscope and Image capture software (Infinity Capture V4.6.0, Lumenera Corporation), taking care to avoid arterioles > 50 µm external diameter. Immunohistochemical staining was quantified using Image-Pro Plus (V.6.3; Media Cybernetics, Silver Spring, MD, USA). We assessed the percent area (% Area) for each case from at least 10 ROI images representing the vascular area stained with COL4 (as % COL4 Area) and to test the quality of the immunoreactvities between individual sections and cases, we ascertained the integrated optical density (IOD). There were no significant differences in IOD values between disease and control samples. There was no obvious relationship between immunohistochemical staining of COL4 or PDGFR-β and length of fixation, or post-mortem interval among any of the groups. The % COL4 area, capillary length and diameters were analysed manually with Image-Pro Plus Analyzer. Throughout histopathological analyses were performed blind to the operator.

### Statistical analyses

Data were analysed using GraphPad Prism and SPSS (V19.0, IBM). Data were first confirmed for normality using the Shapiro–Wilk test. Differences between means of groups were first tested using one-way ANOVA followed by Tukey’s post-hoc test or Kruskal–Wallis H test where appropriate. Linear correlations between age and COL4-positive pericyte numbers per COL4 area (mm^2^) were performed using the Pearson’s correlation [13]. Differences were considered significant with P value less than 0.05 and data are presented as mean ± SEM.

## Results

### Clinicopathological characteristics of the sample

Mean age of older controls and all disease groups was on average 20 years greater than that of young controls (Table 1). Where available, MMSE and CAMCOG scores showed subjects had evidence of dementia at least 6 months prior to death. Compared to both young and old controls, there was significant neurodegenerative or vascular pathology across all the disease groups. Total vascular pathology scores in PSND, PSD, AD, VaD and Mixed subjects was nearly 1.6–2.0 fold higher than old controls (*P* < 0.05). There was greater burden of neurodegenerative pathology in terms of Braak, CERAD and ABC scores in AD and Mixed (AD + VaD) subjects compared to all other groups (*P* < 0.05). The WML and WM/vascular lesion scores were also greater in PSND and across all dementias compared to controls (*P* < 0.01). Notably, there were no significant differences in any of the neurodegenerative pathology staging results between the PSND and PSD subjects (Table 1).

### Pericyte cell bodies in capillaries

**Fig. 1:**
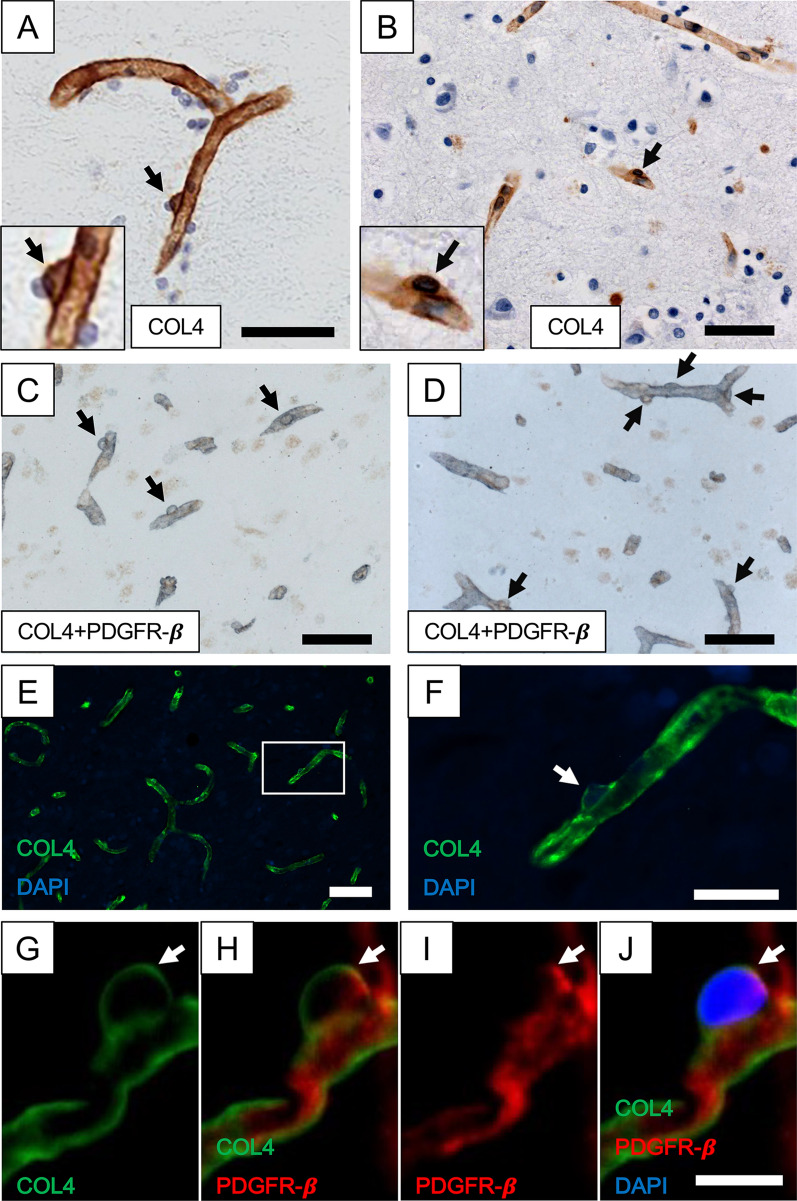
Capillaries with pericytes in the frontal cortex. A and B, Cerebral cortical capillaries immunostained with COL4 from a 95-year-old female PSND subject. Pericytes (black arrows) were identified by the morphology of ‘protrusion’ from the capillary walls surrounded by the COL4-positive membrane and separated from vascular lumen by COL4-positive basement membrane. Insets showing detailed structures of capillary pericytes at higher magnification. C and D, The cortical capillary networks immunostained with COL4 (blue/grey) and PDGFR-β (brown) were similar in both layers III and V [19]. COL4 and PDGFR-β double-positive cells (black arrows) are likely to be pericytes. E and F, Immunofluorescent staining with COL4 (green) and DAPI (blue) in the cerebral cortex, representing a pericyte (white arrow). G-J, Another segment of capillary immunostained with COL4 (green), PDGFR-β (red) and DAPI (blue). Nucleated pericytes double positive for COL4 and PDGFR-β (white arrow) are clearly visible. Images C-D were derived from an 81-year-old female with PSD, E–F, from a 74-year-old VaD and G-J from a 78-year-old PSND subject. Scale bars; A-E = 50 µm; F = 20 µm; J = 10 µm

As previously noted in the frontal white matter, COL4 immunopositive protrusions or “bumps” on segments of cortical microvessels were characterised as cell bodies of capillary pericytes (Fig. 1). Their precise abluminal location and being enveloped by COL4 immunostained basement membranes clearly differentiated them from luminally located endothelial cells (Fig. 1a, b, e, f). In cortical capillaries of 5-7 µm diameter, cell bodies of pericytes were observed to be localised at an approximate distance of 3–5 per mm length in the microvascular network of normally ageing subjects. Double immunostained tissue sections of the frontal cortex showed there was distinct overlap between COL4 and PDGFR-β immunoreactivties, indicating that COL4-positive “protrusions” were pericyte somata (Fig. 1c, d). They were negative for specific markers of the endothelium such as GLUT1 but positive for laminin, another basement membrane marker, which could be used to identify pericyte somata (data not shown). Upon double immunofluorescence with COL4 and PDGFR-β antibodies, we confirmed pericyte cell processes emanating from nucleated cell bodies were positive for PDGFR-β immunoreactivity (Fig. 1g–j). Our observations also confirmed PDGFR-β immunoreactivity was largely restricted to pericyte processes on capillaries and in the virtual absence of αSMA imunoreactivity (< 0.1% in 50 capillaries).

**Fig. 2:**
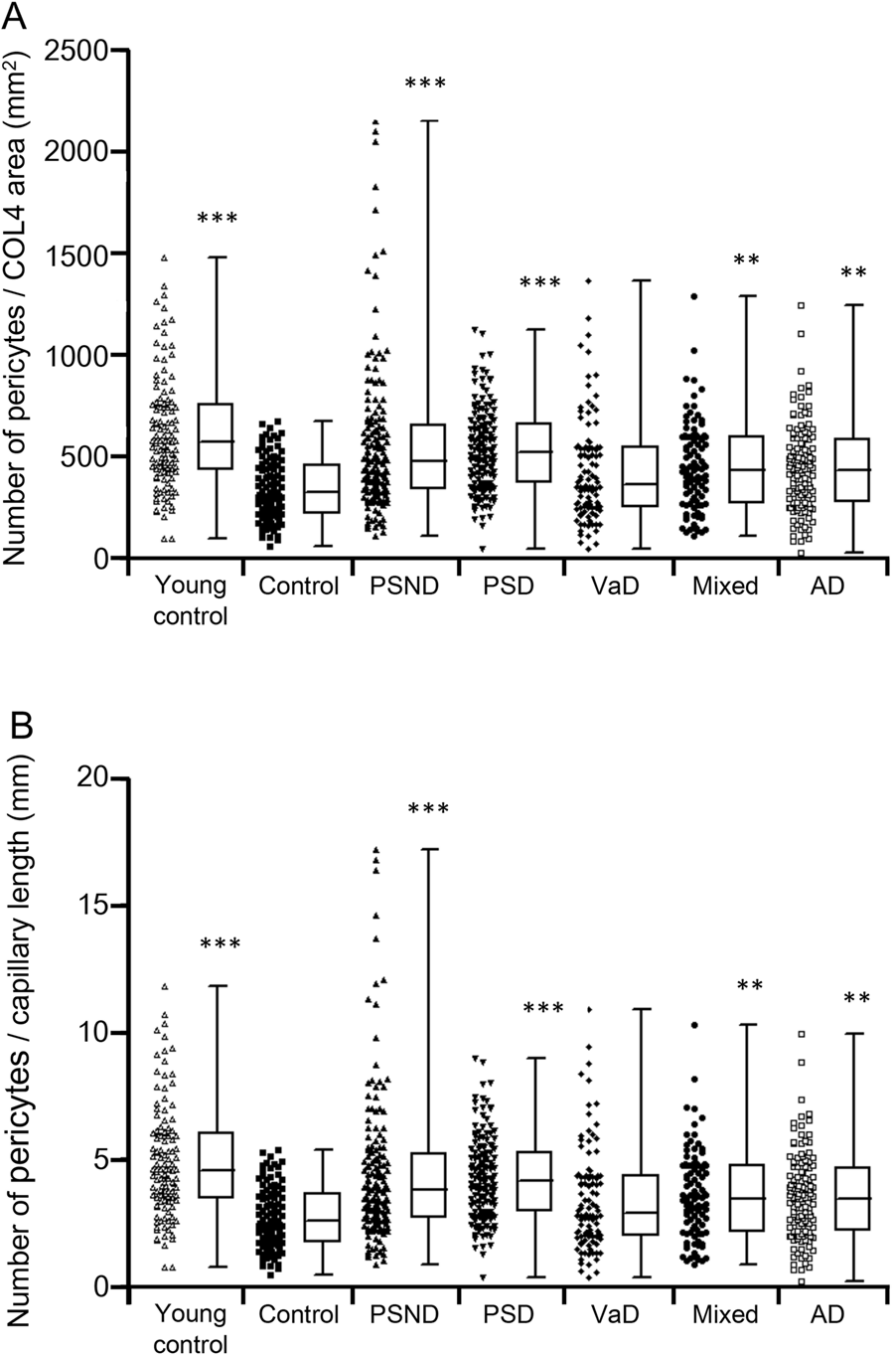
Quantification of frontal cortical pericytes in dementias and ageing controls. A-B, Individual data points and box plots showing number of pericytes per COL4 area (mm^2^) (A) and per unit (mm) capillary length (B). Dots demonstrate distribution of pericytes in all the cases and controls whereas box plots represent mean distribution of pericytes within cases. Per Methods, pericytes were determined in capillaries within regions free of infarcts or CAA. Mean pericyte numbers were increased in PSND, PSD, Mixed and AD compared to older control subjects (***P < 0.001 Control vs PSND and PSD; **P < 0.01 Control vs Mixed and AD). The mean numbers were also greater in young controls compared to older controls. (***P < 0.001). VaD group showed similar pericyte number to controls (*P* > 0.05)

Given our previous quantitative data in the frontal white matter in different dementias, we focussed on the frontal cortex, particularly the medial and dorsolateral region, which is associated with pyramidal cell atrophy across dementias [19]. Quantification of COL4 immunostained pericyte somata with clear nuclei in the frontal cortex in normal young controls (Fig. 2) showed the overall mean (± SEM) densities to be 613 ± 26 per COL4 area mm^2^ and 5.2 ± 0.2 mm capillary length. These estimates in older controls were 340 ± 15 and 2.9 ± 0.1 per COL4 area mm^2^ and mm capillary length, respectively. Thus, there were differences in numbers between young and older controls with a reduction of ~ 45% in older controls (*P* < 0.001) (Fig. 2). However, we did not observe any obvious age-related differences in the size or shape of pericyte somata by light microscopy. Consistent with our previous report [22], we also found that the % COL4 immunoreactive capillary area was increased by 42% in older compared to young controls (Additional file 1). This may reflect increased capillary density as well as thickening of the basement membranes in ageing that could modify pericyte densities [22].

We next quantified pericyte cell bodies within cortical capillaries across different dementias characterised by variable degrees of vascular and neurodegenerative pathologies (Fig. 2). We found that irrespective of the method used for calculation i.e., numbers of pericyte cell bodies per COL4 mm^2^ area or cell densities per mm capillary length, pericytes were significantly greater in the dementia groups including PSD, AD and Mixed groups compared to similar age older controls (Fig. 2). Surprisingly, there were no significant differences in frontal cortical capillary pericytes density between the PSD and PSND groups (*P* > 0.05). Thus, although pericyte numbers were decreased in older controls compared to young controls (*P* < 0.001), they were not decreased in PSND, PSD, Mixed and AD groups compared to the older controls (*P* > 0.05). To negate whether global atrophy or diffuse neocortical ribbon reductions might have affected the observed results, we found no evidence for differences in the total Z scores between PSND and PSD subjects compared to controls. Thus, the total Z scores for cortical atrophy in PSND and PSD groups were 0.12 and 0.22, respectively compared to those in controls was—0.02 (*P* > 0.05).

We also noted there were no substantial changes in the measured % COL4-positive immunostained area in any of the dementia types or post-stroke survivor groups compared to older controls (> 0.05). This was remarkably consistent with no change across these dementias compared to older age controls in our previous independent study [22]. We verified that 3D-stereological assessment of capillary length densities (L_v_) and identified by GLUT1 were also not significantly different across the dementias compared to older controls (Table 1). We previously showed GLUT1 and COL4 immunostained profiles are closely related although there is endothelial thinning and basement membrane thickening in some dementias. However, GLUT1 L_v_ followed a similar pattern to COL4 changes, predominantly labelling capillaries [9, 22]. There was also no trend between any of the ABC scores and numbers of pericyte somata within the dementia groups (*P* > 0.05). This was likely because amyloid β and neurofibrillary pathology had reached the ceiling.

**Fig. 3:**
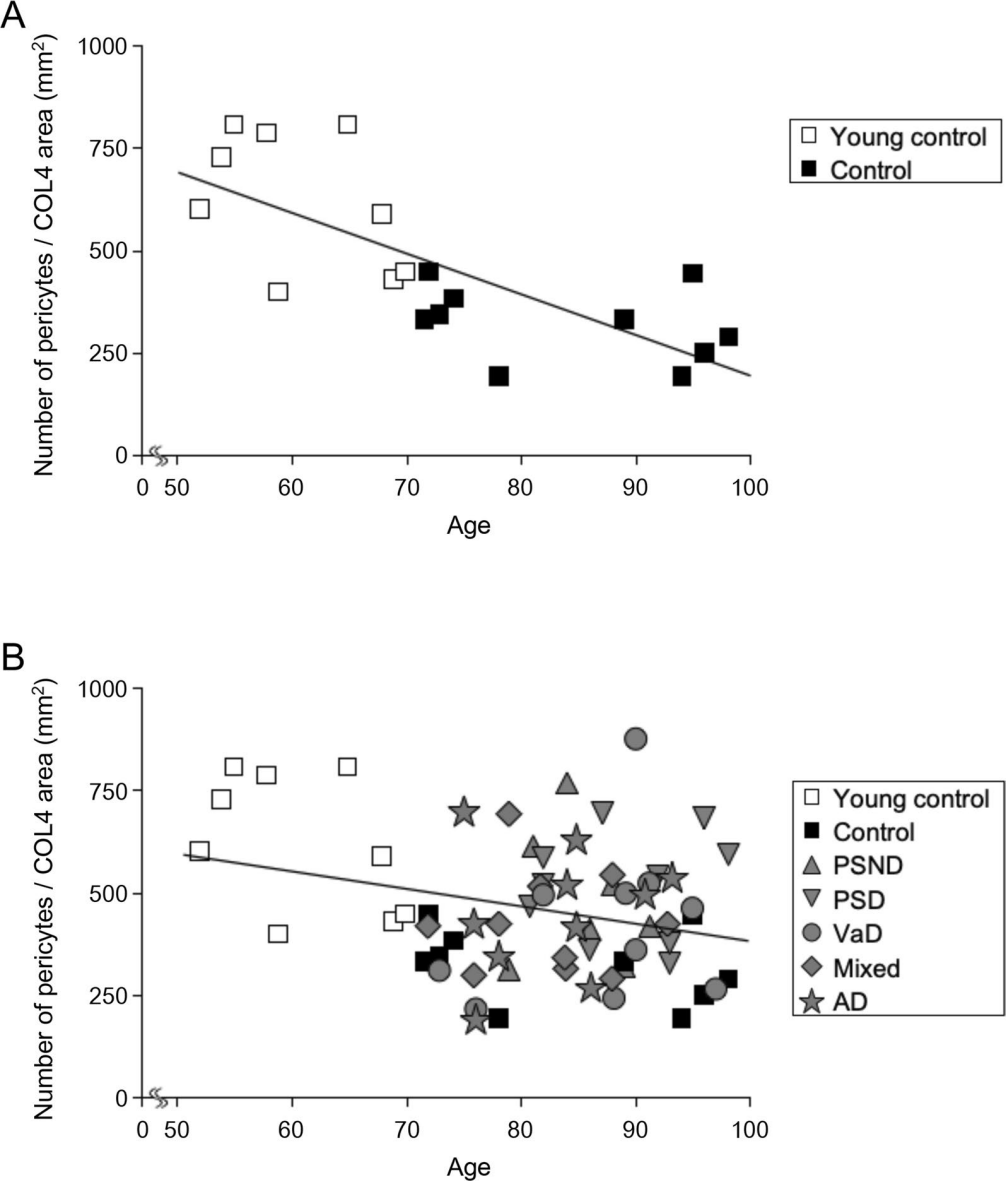
Correlation of number of pericytes per COL4 area and age in the frontal cortex. A, Scatter plot showing negative correlation between number of pericytes per COL4 area (mm^2^) and age amongst young control and older control (Control) subjects, suggesting ageing effects on pericytes numbers in the cerebral cortex (Pearson’s *r* =  − 0.73, *P* = 0.0003). B, Number of pericytes per COL4 area (mm^2^) and age in all dementias and control subjects were also negatively correlated (Pearson’s *r* =  − 0.28, *P* = 0.02), implicating pericytes tend to be reduced with ageing in the total sample indicating an ageing effect in disease groups

In further analysis, we correlated pericyte cell body numbers per COL4 area mm^2^ against age of all normal control subjects and those of all controls and dementia groups (Fig. 3a and b). We found negative correlation with age amongst controls ((Pearson’s *r* = −0.73, *P* < 0.001) and controls and dementia groups (Pearson’s *r* = −0.28, *P* = 0.02) suggesting fewer pericyte somata were apparent in older age in controls although % COL4 immunoreactive capillary area was increased between young and older controls (Additional file 1). However, correlation analysis limited between pericyte soma numbers per COL4 area mm^2^ and age of different disease groups including PSND, and PSD did not show a significant trend (*P* > 0.05).

## Discussion

In accord with our previous study on the underlying white matter [16], we estimated numbers of nucleated pericytes on cortical capillaries rather than pericyte coverage in the frontal cortex across several dementias with vascular and neurodegenerative pathologies. Using conventional immunohistochemical (and immunofluorescence) methods, we found that pericyte cell density was approximately 2–threefold greater in the cortex than the underlying frontal white matter. The differences in pericyte densities are also consistent with the differential densities of capillaries in cerebral cortex and underlying white matter we have reported on previously [22]. We estimated that in the frontal cortex there are 5–6 pericyte cell bodies per mm capillary length or approximately 600 pericyte per COL4 area mm^2^ in normal 46–65-year-olds. Our estimates of pericyte densities in human cortex are consistent with the mid-capillary region of the mouse cortex [5] but are inconsistent with the 2000–3000 pericyte number per mm^2^ vascular area reported previously in human frontal cortex and the hippocampus [44]. We are also confident that our results are not confounded by changes in perivascular microglia or macrophages, which could be mistaken for pericyte cell bodies. While macrophages have different shapes, in a separate study, we found that perivascular macrophages along capillary profiles were not altered across the dementias compared to PSND or similar age controls [25] (Ameen-Ali K et al., unpublished observations). However, the most interesting observation here is that we found an age-related loss of pericyte cell bodies in the frontal cortex. This means that increasing age appears to have a stronger effect on cortical pericyte cell bodies in capillaries than any disease pathology did, either neurodegenerative or vascular of origin. Our observations suggest within the disease groups frontal cortical pericytes were overall preserved [18].

In principle, our observations on age-related pericyte loss are consistent with a previous ultrastructural study on neurosurgical biopsies [46]. They showed that pericyte cell area in capillaries of the frontal and temporal cortex was substantially reduced (> 50%) in 80-year-olds compared to 20-year-olds. In an earlier experimental study [4], age-dependent vascular damage in pericyte-deficient mice was shown to precede neuron degenerative changes accompanied by inflammatory responses and learning and memory impairment. They suggested that pericyte loss results in a progressive age-dependent vascular-mediated neurodegeneration. Previous studies have suggested that while ablation of a single pericyte soma in rodents does not affect focal BBB function [5], the absence of pericytes induces microvessel leakage and microvessel regression [36, 50]. Taken together, the loss of pericytes with age suggests disturbed neurovascular unit and more importantly decreased ability of the BBB to precisely compensate for transient leaks in the elderly. We are not aware of other similar ageing study as ours that could verify our observations.

We also found that cortical pericyte somata were either preserved or marginally increased in different dementias characteristic of neurodegenerative and vascular pathologies. The observations were consistent irrespective of the denominator used to express the results. In contrast with what might be predicted albeit expressed as pericyte coverage [30, 34, 35, 39], we did not observe disease related loss of pericytes in the frontal cortex. These observations are much unlike those in the underlying frontal white matter assessed in the same large coronal sections as the cortex [16, 22]. This indicates cortical pericyte cell numbers are largely preserved or tended to increase in vascular as well as AD and Mixed dementia. We further observed that pericyte cell bodies were increased in post-stroke survivors who did not develop dementia (PSND group) suggesting that even remote vascular changes may increase or remodel the capillary network. These observations are, however, inconsistent with previous studies in which PDGFR-β immunoreactivity was used to assess pericytes [36]. While pericyte cell bodies are preserved or appear immobile [5], it is not unlikely that cell processes or extensions are altered due to energy demand, local perfusion and tissue changes or even proteinaceous toxicity [12, 34, 40]. It is plausible that pericyte cell populations are constantly changing in tandem with angiogenesis, restructuring and microvascular modelling during chronic disease. Our prior observations (Kalaria R et al., unpublished) have shown that markers of angiogenesis such as Ki67 and proliferating cell nuclear antigen seldom labelled endothelial cells in ageing brains. Thus, our findings here do not necessarily reflect a robust angiogenic process. However, the consequence of preserved or even increased pericytes in the context of ischemic injury or neurodegenerative pathology may indicate the presence of microvascular anomalies. Previous experimental studies showed the overexpression of pericyte markers could occur due to impaired revascularization in retinopathy [17] or due to vascular instability during vascular development [8].

Our observations in AD on generally preserved pericyte numbers in the frontal cortex are in contrast with pericyte coverage measured by PDGFR-β immunoreactivity in the prefrontal cortex [30, 36]. They found losses of pericyte coverage associated with accumulation of AD pathology both amyloid β and neurofibrillary tangles [30]. In the study of Miners et al. [34] in which PDGFR-β reactivity was assayed by ELISA as surrogate of pericytes, PDGFR-β reactivity was significantly decreased in AD subjects in the precuneus of the parietal lobe. While there may likely be regional differences in cell bodies, it is possible that surface areas of pericyte cell processes are retracted or reduced in the parietal cortex in AD, but this may not reflect a change in the number of cell bodies. Our results in AD are, however, remarkably in agreement with the recent study in which 3D-stereological quantification showed increased capillary density with largely preserved pericytes in the frontal cortex [18]. Consistent with our observations, the study suggested that cortical pericyte demise is not characteristic of AD pathology although it may be different in the hippocampus [30].

Studies showing lower expression of PDGFR-β immunoreactivity in AD may be explained by increased retraction or atrophy of pericyte cell processes that had not yet lost their nuclei. Previous studies [34, 40] have further suggested that amyloid β is likely directly toxic to pericytes. Our results do not appear to promote the role of soluble or insoluble amyloid β in pericyte degeneration at least in the frontal cortex in AD or Mixed dementia. In addition, we previously reported that both PSD and PSND groups had similar amyloid β load [1] and pericyte numbers were similar yet capillary densities were greater between these groups and high amyloid β load bearing dementias. Thus, our results argue for a different mechanism associated with pericyte changes that may impact on processes but not the cell soma in the cerebral cortex [36]. As far as we could discern, these findings were not influenced by tissue shrinkage or atrophy due to the presence of other pathologies at least in the PSD cases where compaction of the capillary network could occur. In addition, increased cortical capillary densities in AD may occur in a region-specific manner as reported by other investigators [9, 30, 43].

This study has some limitations. First, we did not assess pericyte numbers in other cortical areas, for example in previously anticipated regions such as the precuneus with greater amyloid β load. We surmised that quantification of other regions required a monumental effort that may not reveal different results given that we previously found that cortical capillary densities were largely unchanged or increased in AD. Second, we did not verify the entire quantitative results by also assessing pericytes using PDGFR-β immunoreactivity as another marker. While we irrefutably demonstrated it labels pericytes, PDGFR-β immunostaining was found to be rather capricious for reliable quantification in large numbers of human post-mortem tissues. PDGFR-β immunoreactivity is a frequently used pericyte marker but it also identifies, albeit diffusely, neurons, myofibroblasts, fibroblasts, vascular smooth muscle cells and endothelial precursor cells [3, 15]. This also means that our results are based on pericyte soma counts and could not be entirely related to PDGFR-β immunoreactivity, which is widely used for pericyte coverage (processes) per se. Indeed, the availability of more specific markers of pericytes would also have been useful to verify our findings on the mechanics of pericyte cell impairment or turnover and determine if PDGFR-β is increased intracellularly as reactive response to tissue and microvascular remodelling.

In summary, we found ageing-related loss of numbers of capillary pericytes in the frontal cortex in cognitively normal individuals. Pericyte cell loss is likely associated with age-related disintegration of the neurovascular unit of the cortex that impairs BBB function. This suggests even if one cell type is aberrant within the neurovascular or even the gliovascular unit [23] there could be sequalae to local permeability and perfusion. However, pericyte numbers were largely preserved or marginally increased in dementia compared to similar age controls. They may be modified along with microvascular or capillary remodelling during vascular or stroke injury in elderly individuals. These observations suggest that changes in tissue perfusion and local cellular needs modify pericyte cell responses in capillaries which likely undergo tissue-specific remodelling during chronic disease.

## Supplementary Information

**Additional file 1: Table** Pericyte counts and COL4 % area in cases and ageing controls .

## Data Availability

The data that support the findings of this study are available on request from the corresponding author. The data are not publicly available due to privacy or ethical restrictions.
