# Supplementary material for: Loss with ageing but preservation of frontal cortical capillary pericytes in post-stroke dementia, vascular dementia and Alzheimer’s disease
Source: Acta Neuropathol Commun. 2021 Aug 2;9:130. doi: 10.1186/s40478-021-01230-6 (PMC8330023; doi:10.1186/s40478-021-01230-6)
Supplement: Supplementary file 1 — Additional file 1: Table Pericyte counts and COL4 % area in cases and ageing controls . [file 40478_2021_1230_MOESM1_ESM.docx]

**Supplementary Table: Pericyte counts and COL4 % area in cases and ageing controls**

|  | **Young Controls** | **Older Controls** | **PSND** | **PSD** | **VaD** | **Mixed** | **AD** |
| --- | --- | --- | --- | --- | --- | --- | --- |
| **Number of pericytes / image** | 15.4 ±0.6 | 12.7 ±0.61 | 15.6 ±0.5 | 16.4 ±0.5 | 11.2 ±0.5 | 12.8 ±0.6 | 12.7 ±0.5 |
| **Capillary density (% COL4 stained area)** | 3.3 ±0.1 | 4.7 ±0.1* | 4.0 ±0.1 | 4.0 ±0.1 | 3.6 ±0.1 | 3.8 ±0.1 | 3.9 ±0.1 |
| **Number of pericytes / COL4 mm^2^ area** | 613 ±26 | 340 ±15* | 563 ±30 | 529 ±18 | 430 ±26 | 444 ±21 | 439 ±19 |
| **Number of pericytes /**  **capillary length (mm)** | 5.2 ±0.2 | 2.9 ±0.1* | 4.8 ±0.3 | 4.6 ±0.1 | 3.7 ±0.2 | 3.8 ±0.2 | 3.8 ±0.2 |

Numbers represent mean values (+SEM) for n=10-12 cases or controls per group. *Significance P<0.05 versus young controls. Percent COL4 area was not different between older controls and any of the disease groups (P>0.05, ANOVA).
